# Cost-effectiveness of brief structured interventions to discontinue long-term benzodiazepine use: an economic analysis alongside a randomised controlled trial

**DOI:** 10.12688/hrbopenres.13049.1

**Published:** 2020-06-03

**Authors:** Dominic Trépel, Shehzad Ali, Simon Gilbody, Alfonso Leiva, Dean Mcmillan, Ferran Bejarano, Ermengol Sempere, Caterina Vicens

**Affiliations:** 1 School of Medicine, Trinity College Dublin, Dublin, D02 PN40, Ireland; 2 Global Brain Health Institute, Trinity College Dublin, Dublin, D02 PN40, Ireland; 3 Global Brain Health Institute, University of California, San Francisco, San Francisco, CA, USA; 4 Mental Health and Additions Research Group, Department of Health Sciences, University of York, UK, Heslington, York,, YO10 5DD, UK; 5 Instituto de Investigación Sanitaria de Palma, Palma, 07120, Spain; 6 Institut Català de la Salut, DAP Camp de Tarragona, Catalunya, Catalunya, Spain; 7 Conselleria de Sanitat Universal i Salut Pública, Paterna Health Care Centre, Valencia, 46010, Spain

**Keywords:** Cost effectiveness analysis, Randomised Control Trial, Benzodiazepine, Brief structured interventions, Discontinue of long-term

## Abstract

**Background: **In Spain, long-term use of benzodiazepine is prevalent in 7% of the population; however, this longer-term use lacks clinical benefits, costs €90million per year and side-effects further add extra cost through adverse health outcomes. This study aims to estimate the cost-effectiveness of primary care services stepped dose reduction of long-term benzodiazepines using either Structured Interview with Follow-up (SIF) or Without Follow-up (SIW), compared to Treatment as Usual (TAU).

**Design: **Cost-effectiveness analysis was conducted alongside randomised control utilizing data from three arm cluster randomized trial.

**Setting: **Primary care.

**Participants: **75 general practitioners were randomised to one of the three arms (TAU, SIW, SIF).

**Measurements:** Cost and Cost per Quality-Adjusted Life Year (QALY)

**Results: **Compared to usual care, providing SIW per participant costs an additional €117.94 and adding patient follow-up, €218.4. As a result of intervention, participants showed a gain of, on average, for SIW 0.0144 QALY (95% CI -0.0137 to 0.0425) and for SIF 0.0340 QALYs (0.0069 to 0.0612). The Incremental Cost Effectiveness Ratio was €8190.28/QALY (SIW) and €6423.53/QALY (SIF). At the Spanish reimbursement threshold (€45,000 per QALY) the chance interventions are cost effective is 79.8% for SIW and 97.7% for SIF.

**Conclusions:** Brief structured interventions to discontinue long-term benzodiazepine use represent value for money, particularly with scheduled follow-up appointments, and would represent a cost-effective investment by the Spanish healthcare to reduce prevalence of long-term use.

## Introduction

Long-term prescription of benzodiazepines has significant negative outcomes, causing significant costs to healthcare and wider society; however, many physicians continue to prescribe these and patients wishing to withdraw rarely receive advice or support ^1^. The prevalence of long-term benzodiazepine use in Europe ^2, 3^ remains high despite policy recommendations to limit their use ^4– 7^.

In Spain, long-term use is extensive, with more than 7% of the population using them regularly ^8, 9^, whereas in the United Kingdom, Germany or Netherlands (where policies aim to restrict use), prevalence of their use is less than 2% ^2, 3^. Based on the cost of long-term benzodiazepine prescription alone, we estimate the cost in Spain is over €70 million per annum and, if the consequences of associated morbidities were considered, the full economic cost to healthcare is much higher ^10^.

Evidence suggests that long-term use has a lack of clinical benefits ^11^, and guidelines clearly recommend prescribing on a short-term basis. Given the implications associated with longer-term use, several programs designed to taper off the use of benzodiazepines have been developed, which have been demonstrated to be effective and safe ^12, 13^. However the uptake and use of such interventions is not widespread. A case for the use of such interventions could be supported by robust evidence of cost effectiveness to show that this was an efficient use of finite healthcare resources. To our knowledge there are no full economic evaluations in this area. Economic evidence on benzodiazepine cessation programs report predominantly how costs vary by level of complexity, as studies have tended to report the absolute costs of programs (as opposed to the incremental cost over Treatment as Usual (TAU)). Full economic evaluations are therefore required ^14, 15^.

A clinical trial in primary care has already been reported, which assesses the effectiveness of two interventions to discontinue long-term benzodiazepine use. The two programs evaluated were found to be more effective than usual care in stopping long-term benzodiazepine use in patients without severe co-morbidity ^16^. Having established that both programs are clinically effective in reducing long-term use, the question then arises as to whether they are also cost-effective.

To our knowledge, this is the first cost-effectiveness analysis of benzodiazepine cessation programs in Spain. To inform policy, we simultaneously examine generic health gains (expressed in Quality-Adjusted Life Years (QALYs)) ^17, 18^ and associated costs to healthcare; this aims to inform the decision-makers by comparing the Incremental Cost Effectiveness Ratio (ICER) against willingness-to-pay threshold ^19– 21^. The clinical trial assessed the effectiveness of two interventions in primary care using ‘discontinuation of benzodiazepine use’ as the primary outcome. The economic evaluation adopted the perspective of the Spanish Health system and Spanish unit costs were applied to the cost of general practitioner (GP) training, per-protocol cost of GP intervention, costs of follow up visit and the cost associated with benzodiazepine use.

## Methods

### Participants and interventions

The clinical trial has been described by Vicens *et al.* (2014) ^16^. Briefly, a three-arm, parallel, multicentre, cluster-randomised trial was carried out in Spain, which aimed to compare the effectiveness of two brief structured, tapering interventions delivered by the GP, to discontinue long-term benzodiazepine use versus a control group that received TAU. The current study presents the economic analysis conducted alongside this randomised controlled trial from a health systems perspective.

The interventions included a structured educational interview with gradual dose reduction either (i) with follow-up visits (Structured Interview with Follow-up - SIF) or (ii) with written instructions (Structured Interview without Follow-up - SIW) instead of a follow-up visit. The initial interventions’ visit in both groups lasted about 20 minutes; SIF arm patients scheduled 12 minutes follow-up appointments every 2–3 weeks until the end of the dose reduction and each appointment had reinforced education, reassured patients regarding withdrawal symptoms and obtained patient agreement for the next step in dose reduction. The patients in the SIW group received written instructions reinforcing educational information at their first and only contact with their GP, along with a tailored gradual dose-reduction until benzodiazepine cessation.

GPs from 21 primary care centres in the three regions of Spain were enrolled and, in total, 75 GPs were randomised to one of the three arms (TAU, SIW, SIF). Patients eligible for the trial were aged 18 to 80 years and had been taking benzodiazepines daily for at least 6 months and did not have a severe mental or physical illness. GPs were randomised once the patients were included, and GPs from the intervention groups were scheduled to a two-hour training session in benzodiazepine withdrawal. GPs of the SIF arm received an additional 30 minutes training in how to proceed in the follow-up visits. Because each GP treated several patients, the GP time cost was spread across patients seen by the GP during this study.

### Resource use and associated unit costs

Evidence in the literature would suggest that long-term benzodiazepine use may have negative health consequences that would have cost consequences to health service. However, we restrict our consideration of cost to consider whether the health gains within the trial period justify the cost of the cessation programs plus subsequent benzodiazepine use.

The costs of the cessation programs include GP training and subsequent patient follow-ups, if any, with the GP for benzodiazepine discontinuation. The cost of the training programs per GP was calculated. The training programs were run in three regions as group GP-delivered workshops and the required resources were considered to calculate the program cost (includes cost of training, trainers’ time, travel and accommodation and all other overhead costs).

Patient resource use (both in terms of training time and follow-up visits) were multiplied by the unit cost of a GP visit to obtain total GP cost. The unit cost of GP visit can vary across Spanish regions and are published in official regional bulletin by each region ^22– 24^. The cost associated with training GPs in SIF and SIW were based on the trainer time (in all cases trainers were GPs, so an hourly rate of €75 per hour was used), travel and accommodation costs by regions where training was provided, and cost associated with administration to organise and provide the workshops.

In Spain, benzodiazepine prescribing is regulated and only formulations with a reference price (published authorised by the Spanish Agency of Drugs and Sanitary products (AEMPS)) are available. The nomenclature web application of the AEMPS ^25^ was used to obtain benzodiazepines cost for this price year. Retrospective levels of benzodiazepine use by participants in the randomised control trial was assessed at baseline, 6 month and 12 months; these consumption patterns were reviewed and confirmed by prescription claims in the clinical records.

Costs are presented in Euros (€) for the price year 2013 and for international comparison, equivalent costs are presented in Pound Sterling (GBP) and US Dollar (USD). Since the follow-up period lasted 1 year, no extrapolation over time was executed, discounting was not necessary. Analysis was conducted using Stata 13.

### Outcomes: mapping generic health state utilities from condition specific measure

As the original trial does not include a measure of individuals’ generic health-state (e.g. EQ-5D), to provide outcomes that have broad policy-relevant application (e.g. QALYs), we evaluated several mapping functions to allow cross walking of a condition-specific measure to obtain a utility score. Specifically, Hospital Anxiety and Depression Scale (HADS) scores were available in the clinical trial, which were used to estimate generic health utility outcomes.

To identify an up-to-date and appropriate method to map clinical trial outcomes onto equivalent generic health states, *HERC Database of Mapping Studies* (Version 3.0 - June 2014) was searched ^26^. Within this resource, we identified that Brazier *et al* (2014) ^27^ systematically reviewed studies with estimation of mapping functions in mentally ill populations. Specifically, the authors critically appraised two studies mapping HADS to generic preference-based measures of health and based on their appraisal, and after comparing post-estimation statistics from each study, we selected to utilise the regression analysis of EQ-5D and HADS ^28^. The mapping algorithm utilises the specific scores from the two subscales for depression (HADS-D) and anxiety (HADS-A) to estimate EQ5D index. The results provide individual utility at each time point, and by applying an area under the curve approach these utility values were used to estimate QALYs ^29^.

### Cost effectiveness analysis

Unadjusted Incremental Cost Effectiveness Ratio (ICER) for each intervention group (SIW and SIF) are calculated against TAU using the following formula:

$$ICER=\frac{{\bar{C}}_{I}-{\bar{C}}_{T}}{{\bar{E}}_{I}-{\bar{E}}_{T}}$$

where C is the costs and E is the effects (as QALYs) in the Intervention (I) or TAU(T).

To estimate the joint distributions of cost and QALYs, non-parametric bootstrapping was conducted on the observed data ^30^. Conventionally, the results of the bootstrap are presented as a scatter plot on the cost effectiveness plan ^31^; however, to jointly present confidence interval around costs and outcomes a confidence ellipse is generated. This jointly indicates the incremental costs and QALYs on the 50%, 75% and 95% confidence intervals, indicating in the CE plane the probability space within which we are confident the true ICER is found.

To evaluate cost-effectiveness of our estimates against willingness-to-pay threshold of a health system decision maker, a range of maximum monetary values were used. To present how the probability of interventions being cost effect increases as decision makers willingness-to-pay increases, a cost effectiveness acceptability curve (CEAC) is generated ^21^.

### Ethical approval

Our study protocol has been approved by the Primary Care Research Committee and the Mallorca Ethical Committee of Clinical Research (IB 1146/09 PI).

## Results

At 12 months, data were available from 523/532 (98.3%) patients. The two structured educational interview programs were found to be more effective than usual care in stopping long-term benzodiazepine use over a 12-month period. The relative risks for benzodiazepine discontinuation were 3.01 (95% CI: 2.03 to 4.46) in the SIW and 3.00 (95% CI: 2.04 to 4.40) in the SIF group compared to usual care.

### Unit cost, resource use and cost estimation

Table 1 presents unit costs associated with benzodiazepine use and, as healthcare costs vary in Spanish regions, region-specific costs utilised to provide the GP cessation program are given within the three study regions.

**Table 1.** Unit costs associated with benzodiazepine use and cessation

	Quantity	Cost (€)	Unit	Reference
**Initial GP visit (by region)**				
Balearic Islands	1	62	Per visit	BIOB 2012
Catalunya	1	40	Per visit	DOGC 2012
Valencia	1	56.39	Per visit	DOGV 2005
**Follow-up visit (by region)**				
Balearic Islands	1	32	Per visit	BIOB 2012
Catalunya	1	40	Per visit	DOGC 2012
Valencia	1	28.76	Per visit	DOGV 2005
Prescribed benzodiazepines ^*^				
Alprazolam 0.5	0.50 mg	2.1	Per month	MSSSI 2014
Diazepam 5	5 mg	1.9	Per month	MSSSI 2014
Lormetazepam	1 mg	2	Per month	MSSSI 2014
Lorazepam	1 mg	1.7	Per month	MSSSI 2014
Zolpidem	10 mg	2.8	Per month	MSSSI 2014
Other benzodiazepine	-	1.81	Per month	MSSSI 2014 (Estimated)

^*^Costs presented here of the commonly prescribed benzodiazepines are the weighted average. However, in the individual-level cost-analysis the specific costs per dose formulation is used

To estimate the costs and cost consequences of implementing the cessation program, mean estimates of the use of healthcare resources associated with benzodiazepine use and cessation are calculated (see Table 2). This indicates the average number of GPs that attended training workshops per region and study group, the number of initial visits GPs provide to long-term users of benzodiazepines, and mean reported contact following initial visit. To indicate the consequence to the health care system, Table 2 indicates the proportion of study sample consuming specific benzodiazepine at initial visit, then at 6- and 12-month follow-up.

**Table 2.** Mean use of healthcare resources associated with benzodiazepine use and cessation

Categories of resource use	SIW		SIF		TAU	
	mean	N	mean	N	mean	N
GP attendance per workshop (N) ^*^						
Balearic Islands	3	3	3	3	3	3
Catalunya	4.5	2	4	2	5.5	2
Valencia	2	3	3	3	1.7	3
Initial visit to the GP for Benzodiazepines Cessation Program ^**^						
Balearic Islands	1	61	1	64	0	62
Catalunya	1	60	1	56	0	76
Valencia	1	47	1	71	0	35
**Contact visits to the GP**						
Balearic Islands	3.298	57	6.651	63	3.576	59
Catalunya	2.424	59	4.727	55	2.135	74
Valencia	4.022	45	8.103	68	2.471	34
**Proportion of sample consuming specific benzodiazepine (over time):**						
**Baseline**						
Alprazolam	0.179	168	0.199	191	0.150	173
Diazepam	0.089	168	0.115	191	0.052	173
Lorazepam	0.304	168	0.346	191	0.312	173
Lormetazepam	0.149	168	0.099	191	0.214	173
Zolpidem	0.119	168	0.147	191	0.150	173
Other Benzodiazepine ^***^	0.250	168	0.168	191	0.208	173
**6 months**
No benzodiazepine	0.459	157	0.382	186	0.146	171
Alprazolam	0.096	157	0.113	186	0.140	171
Diazepam	0.096	157	0.177	186	0.070	171
Lorazepam	0.134	157	0.108	186	0.135	171
Lormetazepam	0.064	157	0.081	186	0.216	171
Zolpidem	0.076	157	0.065	186	0.117	171
Other Benzodiazepine ^***^	0.134	157	0.118	186	0.263	171
**12 months**
No benzodiazepine	0.452	168	0.450	191	0.150	173
Alprazolam	0.101	168	0.105	191	0.145	173
Diazepam	0.077	168	0.126	191	0.087	173
Lorazepam	0.149	168	0.188	191	0.243	173
Lormetazepam	0.077	168	0.073	191	0.197	173
Zolpidem	0.077	168	0.042	191	0.098	173
Other Benzodiazepine ^***^	0.119	168	0.126	191	0.277	173

^*^See *Extended data* ^32, 33^ ^**^Mean initial visit to GP are informed by the ‘per-protocol’ approach in that each treatment group specified the incremental GP visit required to provide specific benzodiazepine cessation program ^***^Given the wide array of benzodiazepines available, this resource use table only reports specific formulations with market share above 5%

Applying unit cost to resource use, Table 3 estimates mean costs associated with the healthcare resources that are required to provide the cessation program and the change in the cost of benzodiazepine in each group (no further extrapolation of cost-consequences beyond cost of benzodiazepine was done). Based on the available information, the mean total cost per patient over a 12 month period was €201.05 for SIW, €301.51 for SIF and €83.11 for TAU ^a^.

**Table 3.** Mean costs of healthcare resources associated with benzodiazepine use and cessation

Categories of resource use	SIW		SIF		TAU	
	Cost	N	Cost	N	Cost	N
**Cost per GP workshop (€)**						
Balearic Islands	468	3	543	3	0.00	3
Catalunya	466	2	541	2	0.00	2
Valencia	462	3	543	3	0.00	3
**Cost of initial visit to the GP by participant**						
Balearic Islands	62.00	61	62.00	64	0.00	62
Catalunya	40.00	60	40.00	56	0.00	76
Valencia	56.39	47	56.39	71	0.00	35
**Cost of follow up visits to the GP by participant**						
Balearic Islands	105.54	57	212.83	63	114.44	59
Catalunya	96.95	59	189.09	55	85.41	74
Valencia	160.89	45	324.12	68	98.82	34
**Cost of benzodiazepine consumed (over time):**						
**Baseline**						
Alprazolam	4.50	168	5.01	191	3.79	173
Diazepam	2.04	168	2.63	191	1.19	173
Lormetazepam	7.29	168	8.29	191	7.49	173
Lorazepam	3.04	168	2.03	191	4.36	173
Zolpidem	4.00	168	4.93	191	5.05	173
Other Benzodiazepine ^*^	5.42	168	3.63	191	4.51	173
**6 months**						
Alprazolam	2.41	157	2.85	186	3.54	171
Diazepam	2.18	157	4.05	186	1.60	171
Lormetazepam	3.21	157	2.58	186	3.23	171
Lorazepam	1.30	157	1.65	186	4.41	171
Zolpidem	2.57	157	2.17	186	3.93	171
Other Benzodiazepine ^*^	2.90	157	2.56	186	5.71	171
**12 months**						
Alprazolam	2.55	168	2.64	191	3.64	173
Diazepam	1.76	168	2.86	191	1.98	173
Lormetazepam	3.57	168	4.52	191	5.83	173
Lorazepam	1.58	168	1.50	191	4.01	173
Zolpidem	2.60	168	1.41	191	3.30	173
Other Benzodiazepine ^*^	2.58	168	2.72	191	6.02	173
Total Cost per Average Patient	201.05	149	301.51	173	83.11	152

^*^Total cost only reports specific formulations (over 5% market share) however in analysis specific unit cost applied (Full list of unit cost available on request from the author (see annex)).

### Health-state utility estimation

Individual HADS scores were used to estimate health-state utility values at each time point. Table 4 presents the unadjusted utilities by treatment group and time point. Applying area-under-the-curve approach and specifying accurate individual time intervals, unadjusted annual QALYs are estimated (see Table 5).

**Table 4.** Unadjusted utilities by treatment group and time.

Treatment group	Mean	(s.d.)	Median	Min	Max	N
**SIW**						
Baseline utility	0.749	0.165	0.775	0.257	1.000	160
6 months utility	0.795	0.152	0.826	0.346	1.000	159
12 months utility	0.817	0.174	0.875	0.224	1.000	155
**SIF**						
Baseline utility	0.731	0.154	0.744	0.273	1.000	184
6 months utility	0.799	0.156	0.834	0.289	1.000	186
12 months utility	0.811	0.166	0.850	0.173	1.000	178
**TAU**						
Baseline utility	0.758	0.158	0.773	0.338	1.000	168
6 months utility	0.784	0.152	0.816	0.346	1.000	171
12 months utility	0.804	0.163	0.834	0.349	1.000	160

**Table 5.** Unadjusted annual QALYs.

Treatment group	Mean	(s.d.)	Median	Min	Max	N
SIW	0.8681	0.2014	0.8819	-0.1066	1.6142	145
SIF	0.8693	0.1804	0.8870	0.3222	1.3249	169
TAU	0.8622	0.1756	0.8707	0.3694	1.1945	149

To control for individual-level variation in baseline utility and to estimate incremental QALYs for the intervention groups, ordinary least squares regression was used with baseline utility included as a covariate ( Table 6 presents the regression outputs). The findings suggest that overall the model explains 56.06% of the variance in QALYs, that baseline utility is a significant predictor of change in QALYs, and the incremental QALY for SIW is 0.0144 (p=0.314) and for SIF is 0.0340 (p=0.014).

**Table 6.** Regression analysis QALY controlling for baseline utility, treatment group.

QALY	Coefficient	Std. Err.		P>t	[95% Confidence Interval]	
Baseline utility	.8658	.0356	24.30	0.000	.796	.9357
SIW	.0144	.0143	1.01	0.314	-.0137	.0425
SIF	.0340	.0138	2.46	0.014	.0069	.0612
Constant	.2020	.0290	6.97	0.000	.1453	.2588

n= 463 R ^2^ = 0.5606

### Incremental cost effectiveness analysis

**Figure 1.**
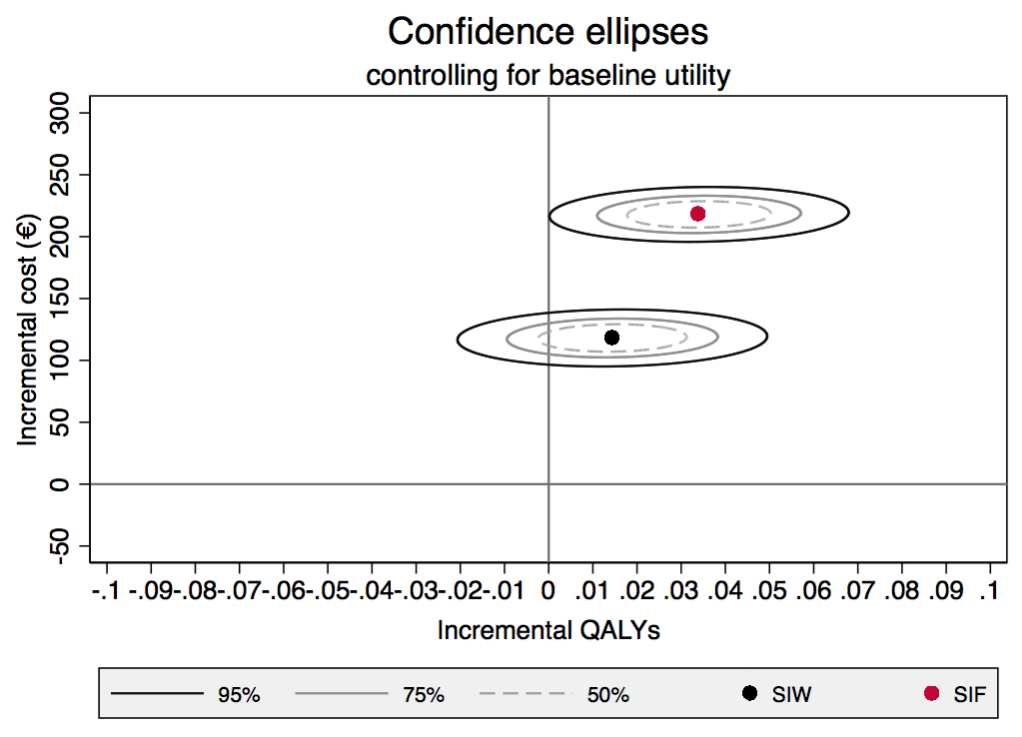
Confidence ellipse controlling for baseline utility.

The regression outputs suggest that baseline utility is a significant predictor overall QALY, so the adjusted coefficients are utilised to calculate point estimate of ICER. This indicates that the ICER for SIW is €8190.28/QALY and for SIF is €6423.53/QALY. However, examining 95% confidence intervals and statistical significance of the regression coefficients for SIW and SIF indicates a degree of uncertainty of the estimated health gain. To further explore the uncertainty surrounding the point estimate, confidence ellipses are generated based on non-parametric bootstrap (see Figure 1).

Examining these confidence ellipses, the shapes suggest that there exists significant variation in the incremental QALY than incremental cost. We can also conclude that at a 95% confidence level we are certain that the two programs will cost more but are less certain about incremental QALYs, particularly for the SIW group.

**Figure 2.**
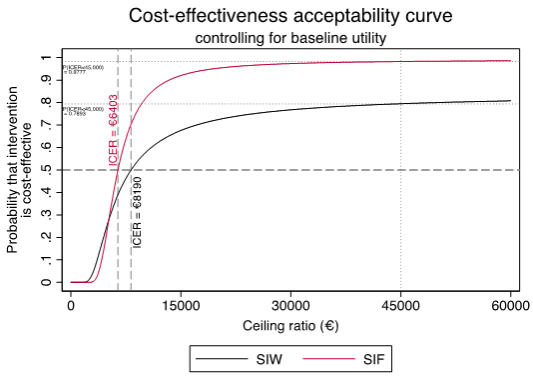
Cost-effectiveness acceptability curve (CEAC) for cost per QALY analysis.

CEAC is generated to show how the probability that interventions will be cost effective as the decision-makers willingness-to-pay is increased (see Figure 2). The ICERs beyond which the interventions become more likely to be cost effectiveness are €8190.28/QALY (SIW) and €6423.53/QALY (SIF). This also illustrates that SIF has a higher certainty of being cost-effective.

At the commonly cited Spanish healthcare decision threshold of €45,000/QALY, the probability that SIW and SIF program being cost-effective was estimated to be P(ICER _SIW_<45,000/QALY)=0.7983 and P(ICER _SIF_<45,000/QALY)=0.9777, respectively.

## Discussion

This study extends beyond a definitive intervention trial with a clinically significant finding and presents within-trial cost-effectiveness analysis of two strategies for benzodiazepine cessation. The incremental cost effectiveness ratios suggest that both forms of interventions may represent value for money as they fall below explicitly cited decision-makers willingness-to-pay threshold ^27^. This study presents findings that suggest that benzodiazepine cessation may be likely to improve individuals overall generic health state.

GP training on managing long-term benzodiazepine cessation is associated with health benefit to patients. Many benzodiazepines are available in Spain and there is no simple way to examine whether the level of potential health gain may be directly associated with withdrawal from products however comparison to usual care find a small but significant incremental cost that appears justified by the expected benefit in each intervention group.

The perspective adopted was that of the healthcare payer (the Spanish Ministry of Health) and the calculation of total cost is based on intervention training costs, GP time and costs of benzodiazepine prescribed. The base-case does not extrapolate a model to indicate the adverse event or risk associated with long-term benzodiazepine use (e.g. falls, fractures, cognitive impairment or premature death). Such extrapolation could augment health-related quality of life (or costs to healthcare) and hypothetically alter ICERs. However, as the cost of intervention justifies health gain, the approach adopted is already justified and, in the absence of a comprehensive measure of resource use, model-based augmentation would require assumptions and may simply raise more questions than answers.

Conversion of HADS score onto associated EQ5D utility was used to provide a means to estimate incremental QALYs. This approach has some limitation. Firstly, Brazier *et al* indicate that the relationship between the EQ-5D and HADS differed across the severity scale, where the concordance between the measures was higher at the less severe end of the scale. Secondly, health state utilities are based on the UK tariff. Finally, the use of HADS scores bases the estimate of generic health solely on changes in anxiety and depression and overlooks other potential domains of generic health (e.g. EQ5D illicit QALYs based on mobility, self-care, usual activities, pain/discomfort and anxiety/depression). In the absence of a preference-weighted measure of generic health (such as EQ5D), this provides an imperfect solution to estimate QALYs.

Nevertheless, analyses based on the currently available information are supportive of a wider implementation of GP training for benzodiazepine cessation in Spain. There does remain a level of uncertainty in the estimated results, as well as limitations in the costs and outcomes utilised and a healthcare decision-maker may alternatively commission further research. The confidence ellipse provides support that there is value in additional information, as it would suggest that the true ICER is most likely to lie in the top right quadrant of the cost effectiveness plane suggesting any further investment (either towards implementation or additional research) will likely represent value for money to the Spanish Ministry of Health.

## Data availability

### Underlying data

Zenodo: Cost-effectiveness of brief structured interventions to discontinue long-term benzodiazepine use: an economic analysis alongside a randomised controlled trial, http://doi.org/10.5281/zenodo.3823846 ^34^.

### Extended data

Zenodo: Supplemental 1: Full list of all benzodiazepine unit costs. Cost-effectiveness of brief structured interventions to discontinue long-term benzodiazepine use: an economic analysis alongside a randomised controlled trial, https://doi.org/10.5281/zenodo.3835494 ^32^.

Zenodo: Supplemental 2: Costs associated with providing training workshops. Cost-effectiveness of brief structured interventions to discontinue long-term benzodiazepine use: an economic analysis alongside a randomised controlled trial https://doi.org/10.5281/zenodo.3835506 ^33^.

Data are available under the terms of the Creative Commons Attribution 4.0 International license (CC-BY 4.0).

## Notes

^a^Average Exchange Rate: GBP/EURO (2012-13): 0.8141; GBP/USD (2012-13): 0.6325 [Sources: http://www.hmrc.gov.uk/exrate/exchangerates-1213.pdf].
