# Supplementary material for: Cost-effectiveness of brief structured interventions to discontinue long-term benzodiazepine use: an economic analysis alongside a randomised controlled trial
Source: HRB Open Res. 2020 Jun 3;3:33. [Version 1] doi: 10.12688/hrbopenres.13049.1 (PMC7372528; doi:10.12688/hrbopenres.13049.1)
Supplement: Peer review history

# Peer review: Reviewer response for version 1

**DOI:** 10.21956/hrbopenres.14145.r27494

**Published:** 2020-07-20

**Authors:** Joachim Marti

**Affiliations:** 1 Centre for Primary Care and Public Health (Unisanté), University of Lausanne, Lausanne, Switzerland

In this paper, the authors conduct a within-trial economic evaluation of brief interventions to discontinue long-term benzodiazepines use in the primary care setting in Spain. Specifically, they performed an incremental cost-effectiveness analysis of structured interviews with or without follow-up vs. usual care and find that both interventions are cost-effective, with ICERs well under the Spanish reimbursement threshold in both cases.

 This is a particularly important topic and evidence is scarce regarding cost-effectiveness in this area. Long-term benzodiazepines use is a public health problem in most developing countries and studies are needed to show value for money of discontinuation strategies. This study therefore makes a valuable contribution to the literature.

 The paper is methodologically sound and follows best practice guidelines in economic evaluation. It is well structured, and the analytical steps and assumptions are transparent. I have however a few comments and suggestions for clarification/possible improvement.

 The abstract needs improvement in terms of style and content. Key information is lacking, especially the details on participants (GPs are mentioned, but brief details on patients included are needed, i.e. age range, definition of long-term use, etc.). Also, check consistency regarding direct (?) costs of long-term benzodiazepines use (figures in the abstract and in the text are not consistent). More generally, the paper would require some editing (e.g. check consistency of tense).

 One important aspect is the definition of costs of benzodiazepines use, which is limited to direct drug costs in the paper, and more generally the scope of costs included. I would recommend clarifying the following statement (especially the last part) in the “Resource use and associated unit costs” section: “However, we restrict our consideration of cost to consider whether the health gains within the trial period justify the cost of the cessation programs plus subsequent benzodiazepine use”. Also it is not fully clear whether GP cost is simply the unit cost of a GP visit multiplied by number of visits, or if broader costs were taken into account (including patient time costs) – there is conflicting information about this in the text. In addition, was the cost of training limited to trainer time or also included opportunity cost for trainees? Finally, the authors explain that estimating the benefits of benzo discontinuation in terms of avoided costs related to adverse events is challenging. This is true but they can be quite confident that their ICERs are conservative and this could be more emphasised in the text.

 In terms of outcomes, mapping is used from HADS to QALYs to derive utility scores. Given the limitations of this approach (that are clearly stated), it could be useful to show results in terms of cost per HADS score change (cost-effectiveness) in addition to cost per QALY (cost-utility), and perhaps other expression of value such as cost per successful discontinuation (needs more details on how this was measured in the trial)?

Is the work clearly and accurately presented and does it cite the current literature?

Yes

If applicable, is the statistical analysis and its interpretation appropriate?

Yes

Are all the source data underlying the results available to ensure full reproducibility?

Yes

Is the study design appropriate and is the work technically sound?

Yes

Are the conclusions drawn adequately supported by the results?

Yes

Are sufficient details of methods and analysis provided to allow replication by others?

Partly

Reviewer Expertise:

Health economics, economic evaluation, addiction, health services research, empirical policy evaluation, health care financing

I confirm that I have read this submission and believe that I have an appropriate level of expertise to confirm that it is of an acceptable scientific standard.

# Peer review: Reviewer response for version 1

**DOI:** 10.21956/hrbopenres.14145.r27496

**Published:** 2020-07-20

**Authors:** Stephen Kinsella

**Affiliations:** 1 Department of Economics, University of Limerick, Limerick, Ireland

- The paper is the first study to evaluate the cost effectiveness of this drug in this setting. Protocols seem to have been followed accurately from the methods section. The CEA seems accurately deployed. Costings at programme level were done and every effort at comprehensiveness seems to have been taken. ICER calculations controlling for baseline utility seem accurate at 95% CI.
- The finding that both interventions are in fact cost-effective at 45k/QALY deserves a little more discussion.
- There are some typos in the manuscript, e.g. discussion, first para, last sentence should read “hospitals’”. Typographical issues I leave to the journal.
- I recommend indexing. 

Is the work clearly and accurately presented and does it cite the current literature?

Yes

If applicable, is the statistical analysis and its interpretation appropriate?

Yes

Are all the source data underlying the results available to ensure full reproducibility?

Yes

Is the study design appropriate and is the work technically sound?

Yes

Are the conclusions drawn adequately supported by the results?

Yes

Are sufficient details of methods and analysis provided to allow replication by others?

Yes

Reviewer Expertise:

Economics.

I confirm that I have read this submission and believe that I have an appropriate level of expertise to confirm that it is of an acceptable scientific standard.
